# The Transcriptome of *Streptococcus pneumoniae* Induced by Local and Global Changes in Supercoiling

**DOI:** 10.3389/fmicb.2017.01447

**Published:** 2017-07-31

**Authors:** Adela G. de la Campa, María J. Ferrándiz, Antonio J. Martín-Galiano, María T. García, Jose M. Tirado-Vélez

**Affiliations:** ^1^Unidad de Genética Bacteriana, Centro Nacional de Microbiología, Instituto de Salud Carlos III Madrid, Spain; ^2^Presidencia, Consejo Superior de Investigaciones Científicas Madrid, Spain; ^3^Departamento de Microbiología, Facultad de Ciencias Biológicas, Universidad Complutense Madrid, Spain

**Keywords:** DNA supercoiling, DNA topoisomerases, fluoroquinolones, global transcription, interactome, novobiocin, seconeolitsine, topological domains

## Abstract

The bacterial chromosome is compacted in a manner optimal for DNA transactions to occur. The degree of compaction results from the level of DNA-supercoiling and the presence of nucleoid-binding proteins. DNA-supercoiling is homeostatically maintained by the opposing activities of relaxing DNA topoisomerases and negative supercoil-inducing DNA gyrase. DNA-supercoiling acts as a general *cis* regulator of transcription, which can be superimposed upon other types of more specific *trans* regulatory mechanism. Transcriptomic studies on the human pathogen *Streptococcus pneumoniae*, which has a relatively small genome (∼2 Mb) and few nucleoid-binding proteins, have been performed under conditions of local and global changes in supercoiling. The response to local changes induced by fluoroquinolone antibiotics, which target DNA gyrase subunit A and/or topoisomerase IV, involves an increase in oxygen radicals which reduces cell viability, while the induction of global supercoiling changes by novobiocin (a DNA gyrase subunit B inhibitor), or by seconeolitsine (a topoisomerase I inhibitor), has revealed the existence of topological domains that specifically respond to such changes. The control of DNA-supercoiling in *S. pneumoniae* occurs mainly via the regulation of topoisomerase gene transcription: relaxation triggers the up-regulation of gyrase and the down-regulation of topoisomerases I and IV, while hypernegative supercoiling down-regulates the expression of topoisomerase I. Relaxation affects 13% of the genome, with the majority of the genes affected located in 15 domains. Hypernegative supercoiling affects 10% of the genome, with one quarter of the genes affected located in 12 domains. However, all the above domains overlap, suggesting that the chromosome is organized into topological domains with fixed locations. Based on its response to relaxation, the pneumococcal chromosome can be said to be organized into five types of domain: up-regulated, down-regulated, position-conserved non-regulated, position-variable non-regulated, and AT-rich. The AT content is higher in the up-regulated than in the down-regulated domains. Genes within the different domains share structural and functional characteristics. It would seem that a topology-driven selection pressure has defined the chromosomal location of the metabolism, virulence and competence genes, which suggests the existence of topological rules that aim to improve bacterial fitness.

## Introduction

The compaction of DNA by up to 1000-fold (Holmes and Cozzarelli, 2000) in the bacterial chromosome, or nucleoid, achieves the optimal condition under which its essential functions – replication, segregation and gene expression (reviewed by Dorman, 2013) – can be reconciled. This compaction is mediated by both the natural supercoiling of the DNA, and by the binding of nucleoid-associated proteins (NAPs) (Wang et al., 2013). NAPs form a functional network that maintains DNA topology by bending, wrapping, bridging and constraining supercoils. Although several NAPs have been characterized in the Gram-negative bacterium *Escherichia coli*, very few have been detected in Gram-positive bacteria, including the human pathogen *Streptococcus pneumoniae* (Dillon and Dorman, 2010). In bacteria, gene transcription is regulated by DNA-supercoiling. This functions as a general *cis* regulator of transcription, and can be superimposed upon other types of more specific *trans* regulatory mechanisms. *cis* regulation can also occur via promoter DNA sequences. Factors acting in *trans* include structural and regulatory proteins. NAPs (structural proteins) target a number of genes (Dillon and Dorman, 2010), while specific regulatory proteins facilitate or inhibit the interaction of RNA polymerase with specific promoter regions (Browning and Busby, 2004). The precision balance of DNA supercoiling is thus modulated by a network of self-regulating factors.

DNA topoisomerases, which are present in all bacteria, are responsible for the maintenance of DNA-supercoiling. These enzymes are classified into two types based on their DNA cleavage pattern: type I, which cleaves only one DNA strand, and type II, which cleaves both. The type II topoisomerases, gyrase and topoisomerase IV (Topo IV), are tetrameric proteins with two subunits: GyrA_2_GyrB_2_ in gyrase, and ParC_2_ParE_2_ in Topo IV. Supercoiling homeostasis is achieved by the competing activities of gyrase and topoisomerase I (Topo I, a type I isomerase) plus IV (Champoux, 2001); gyrase introduces negative supercoils into DNA (Gellert et al., 1976), Topo I relaxes DNA, and Topo IV both relaxes DNA and participates in chromosome partitioning (Kato et al., 1990). *S. pneumoniae* (the pneumococcus) has a relatively small genome (∼2 Mb compared to ∼4.6 Mb for *E. coli*) rich in AT (60%), that carries genes for all three of the above enzymes. These characteristics are shared by other pathogens of the genus *Streptococcus*, including *S. pyogenes* and *S. suis*.

*Streptococcus pneumoniae* is the primary cause of community-acquired pneumonia, meningitis, bacteremia, and otitis media in children. Worldwide, 1 million children under 5 years of age die every year of pneumococcal infections (World Health Organization, 2007). The use of the pneumococcal 7-valent conjugate vaccine, which covers the serotypes most often associated with resistance to antibiotics, has achieved a decline in the incidence of invasive pneumococcal disease (Whitney et al., 2003; Kyaw et al., 2006) and a reduction in penicillin resistance rates (Kyaw et al., 2006; Pilishvili et al., 2010). However, serotypes not included in the vaccine soon emerged, highlighting the limitations of anti-pneumococcal prophylaxis (Moore et al., 2008; Fenoll et al., 2009).

The post-genomic age is beginning to provide answers to questions regarding how chromosomes are topologically organized, and how this organization influences bacterial evolution. Several degrees of organization in bacterial chromosomes have been observed, based on size (for a recent review see Badrinarayanan et al., 2015). Macrodomains are found at the megabase-size range. *E. coli*, for example, has four macrodomains: Ori (origin of replication), Ter (terminus of replication), Left, and Right, plus two less-structured regions flanking the Ori macrodomain (Espeli et al., 2008). Macrodomains may be maintained by specific proteins, such as the macrodomain Ter proteins (MatPs) that bind, as the name suggests, to specific sites in the Ter macrodomain (Dupaigne et al., 2012). However, no such proteins stabilizing the other macrodomains have been identified, and MatP proteins are found only in enteric bacteria. Non-homologous proteins may therefore take on similar roles in other bacteria. Supercoiling domains are found at the kilobase range. These are isolated loops that coil up around themselves; proteins at their bases help to topologically isolate the looped DNA. These loops were initially detected in electron micrographs of lysed *E. coli* cells (Kavenoff and Bowen, 1976). Later studies estimated the number of supercoil domains by assessing the numbers of nicks required to fully relax the chromosome. From these experiments it was estimated that the *E. coli* chromosome contains about 40 domains of around 100 kb (Worcel and Burgi, 1972; Sinden and Pettijohn, 1981). Studies in *Caulobacter crescentus* suggested domains ranging in length from 30 to 420 kb (Le et al., 2013). In *Salmonella enterica*, these domains were estimated to be 20 kb long by taking into account the site-specific recombination events that occurred between chromosomal sites distant from one another (Higgins et al., 1996). Later, transcriptional data predicted sizes of ∼10 kb for *E. coli* (Postow et al., 2004). Controversy regarding the size and definition of domains remains, perhaps as a consequence of the different methods being used in their calculation.

**FIGURE 1:**
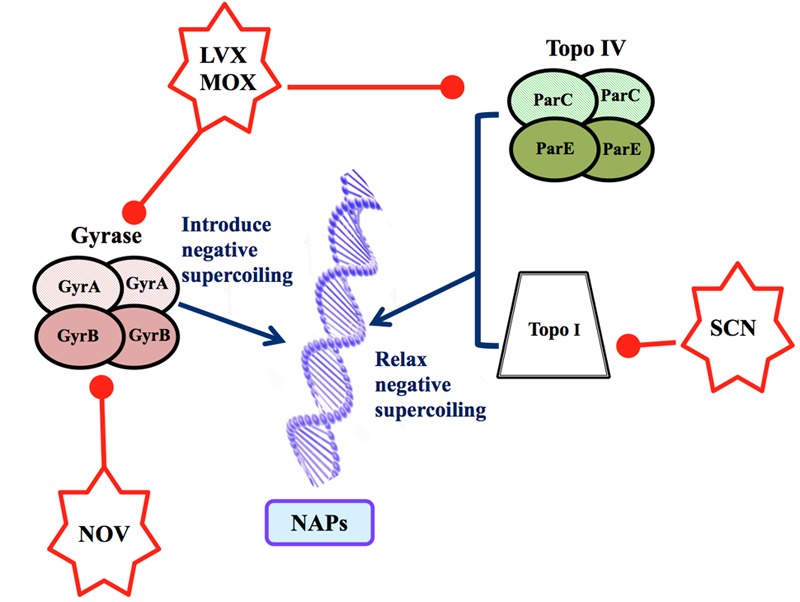
Factors determining the topology of the *Streptococcus pneumoniae* chromosome. The level of supercoiling is controlled by three DNA topoisomerases. Gyrase (GyrA_2_GyrB_2_) is inhibited by LVX, MOX (which inhibit GyrA) and NOV (which inhibits GyrB). Topo IV (ParC_2_ParE_2_) is inhibited by LVX and MOX (which inhibit ParC). Topo I, a monomer, is inhibited by SCN. The topological organization of the chromosome depends on the level of DNA supercoiling and on the presence of NAPs.

The availability of drugs against all the topoisomerases of *S. pneumoniae* (**Figure 1**) has helped in determining the existence of chromosomal domains. This review summarizes the transcriptomic alterations induced by these agents, and how these changes can be interpreted to provide definitions of the chromosome domains in this bacterium. Changes induced by the clinically used fluoroquinolones (FQs) levofloxacin (LVX), and moxifloxacin (MOX) are first considered, followed by those that occur concomitantly with a global change in supercoiling, as induced by novobiocin (NOV, an inhibitor of the gyrase B subunit) and seconeolitsine (SCN, an inhibitor of Topo I). Overall, these studies reveal the *S. pneumoniae* genome to be organized into topology-reacting gene clusters, or supercoiling domains. The conservation of the location of these domains in the *Streptococcus* genus, and their enrichment for specific functions, suggests the existence of topological rules that aim to improve fitness via tight physiological feedback.

## Control of Transcription by Local Changes in Supercoiling

Strains of *S. pneumonia*e resistant to antibiotics that act on the cell wall (beta-lactams) and on protein synthesis (macrolides) have proliferated in the last 30 years (Jacobs et al., 2003; Liñares et al., 2010). Consequently, pneumococcal infections are nowadays fought with LVX and MOX, which inhibit DNA topoisomerases. FQs target the type II DNA topoisomerases gyrase and Topo IV. Their mechanism of action involves the formation of DNA-FQ-topoisomerase complexes, which sterically inhibit replication and transcription and the subsequent generation of detrimental double-stranded DNA breaks (Drlica et al., 2008). Bacterial survival depends on the resolution of these breaks. Reactive oxygen species (ROS), such as superoxide anions, hydrogen peroxide and hydroxyl radicals contribute to FQ-mediated cell death via a protein synthesis-dependent pathway (Wang et al., 2010). This observation is consistent with the general model explaining the lethality of bactericidal antibiotics, which attributes a role to ROS generated via the Fenton reaction. The original reports supporting this model based their conclusions on the use of microarrays to study the transcriptional response to the inhibition of *E. coli* GyrA by an FQ or the peptide toxin CcdB. Under these conditions, global transcription was altered. In addition to the up-regulation of SOS damage response genes, genes related to superoxide stress, iron-sulfur cluster synthesis and iron uptake were up-regulated too (Dwyer et al., 2007). ROS production was also observed with a variety of bactericidal antibiotic families, in addition to FQs, each with a different intracellular target (reviewed by Dwyer et al., 2015). However, the intervening pathways lying between the initial antibiotic-target interaction and ROS formation have yet to be fully characterized.

**FIGURE 2:**
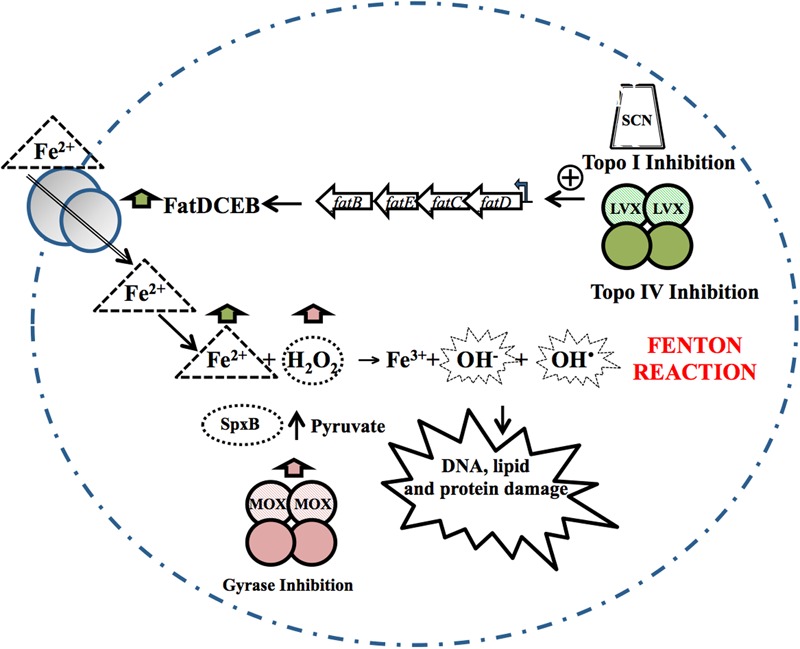
Oxidative damage cell death pathway. The inhibition of Topo IV by LVX, or of Topo I by SCN, causes a local increase in supercoiling, resulting in the up-regulation of the *fatDCEB* operon. The consequent increase in the iron transporter it encodes causes an increase in intracellular Fe^2+^. MOX alters the transcriptome, up-regulating genes from several metabolic pathways involved in the production of pyruvate. Pyruvate can then be converted by pyruvate oxidase (SpxB) into hydrogen peroxide (H_2_O_2_). Fe^2+^ and H_2_O_2_ are the substrates of the Fenton reaction. The Fenton reaction renders hydroxyl radicals, which oxidatively damage DNA, proteins and lipids. Taken from Ferrándiz and de la Campa (2014), with modifications.

The treatment of *S. pneumoniae* with FQs involves causing double-stranded breaks in the bacterial chromosome (Ferrándiz et al., 2016b), and as in other bacteria this requires active protein synthesis (Brito et al., 2017). Treatment with LVX or MOX (Ferrándiz and de la Campa, 2014; Ferrándiz et al., 2016b) is reported not to alter the level of global supercoiling. Nor are changes in supercoiling observed in *E. coli* exposed to oxolinic acid (Snyder and Drlica, 1979), although changes have been observed in the latter after treatment with the FQ norfloxacin (Peter et al., 2004). These differences might be attributable to species-dependent affinities of each drug for Topo IV or gyrase. For instance, Topo IV is the primary target of most FQs in Gram-positive bacteria, including *S. pneumoniae*, with gyrase a secondary target (Janoir et al., 1996; Muñoz and de la Campa, 1996; Tankovic et al., 1996; Fernández-Moreira et al., 2000). In contrast, in Gram-negative bacteria, including *E. coli*, gyrase is the primary target. At the LVX concentrations used in *S. pneumoniae* experiments, only Topo IV would have been inhibited, and no global change in supercoiling would be expected. However, at the MOX concentrations used, both gyrase and Topo IV would have been inhibited, suggesting that the inhibition of their opposing activities preserved the net level of supercoiling. Nevertheless, local topological changes are predictable in both cases and these would produce alterations in the transcriptome. Indeed, FQs induce a transcriptional response in *S. pneumoniae*, in which the differentially expressed genes (DEGs) account for 5.2 and 6.5% of the genome for LVX and MOX, respectively. In this bacterium, which lacks a proper SOS-like system, activation of the competence regulon has been reported with both FQs (Ferrándiz and de la Campa, 2014; Ferrándiz et al., 2016b), supporting the idea that competence is a general stress response in *S. pneumoniae* (Prudhomme et al., 2006). In addition, both LVX and MOX induce transcriptional alterations, which, although different, ultimately stimulate the Fenton reaction, increasing ROS accumulation and contributing to cell death (Ferrándiz and de la Campa, 2014; Ferrándiz et al., 2016b). Although *S. pneumoniae* is a facultative anaerobe, the increased lethality of FQs mediated by an increase in ROS fits with the antibiotic lethality model proposed for aerobic bacteria (Dwyer et al., 2007, 2014; Kohanski et al., 2007; Wang and Zhao, 2009). Via local supercoiling changes, the response to LVX specifically triggers the up-regulation of the *fatDCEB* operon. This causes an increase in intracellular iron, and in turn, a shift in the Fenton reaction toward the production of hydroxyl radicals. With MOX, the response leads to the up-regulation of the glycolytic pathway, with a noticeable increase in pyruvate and a subsequent increase in hydrogen peroxide (**Figure 2**). The different alterations in the patterns of gene expression induced by LVX and MOX are due to local changes in supercoiling, which are dependent on whether Topo IV (LVX) or both Topo IV and gyrase (MOX) are inhibited.

Since both Topo IV and gyrase produce double-stranded breaks in the DNA when bound to FQs, the differential transcriptional alterations caused by these drugs might also be related to subtle, yet important, differences in sequence recognition (Leo et al., 2005), which are themselves affected by DNA supercoiling and bending (Arnoldi et al., 2013). Sequence recognition mediated by local supercoiling levels might explain the unique distribution of genes affected by LVX or MOX. In addition, the location of FQ-topoisomerase complexes relative to the replication forks, which is different for gyrase and Topo IV (Postow et al., 2001), may be involved in their different transcriptional outcomes.

## Control of Transcription by Global Changes in Supercoiling

### Response to Relaxation Caused by the Inhibition of Gyrase

**FIGURE 3:**
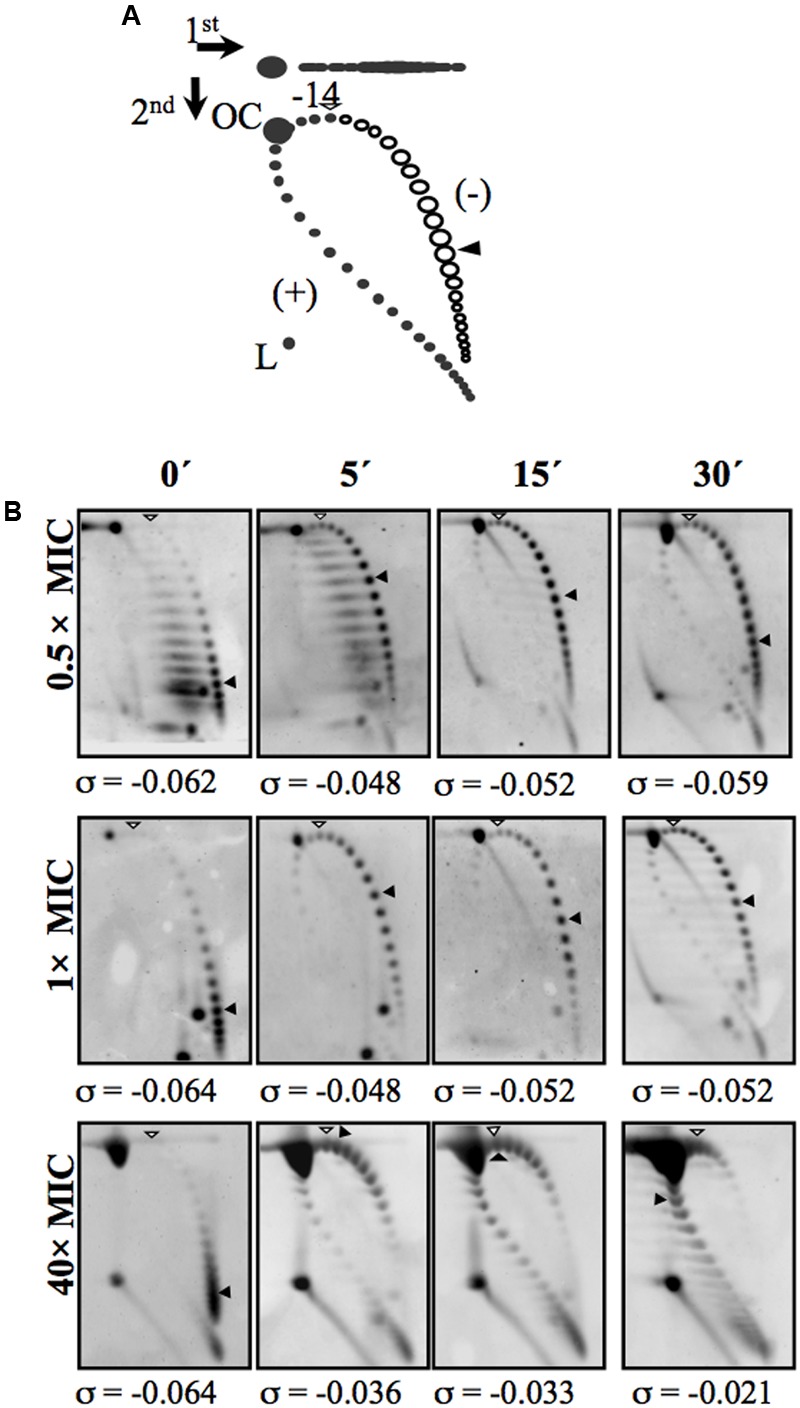
Treatment with the GyrB inhibitor NOV causes relaxation and subsequent recovery of supercoiling levels. **(A)** Diagram showing plasmid pLS1 topoisomer distribution after two-dimensional electrophoresis in agarose gels run in the presence of 1 and 2 μg/ml chloroquine in the first and second dimensions, respectively. Arrows at the top left corner indicate the running direction of the first and second dimensions, respectively. OC, open circle; L, linear form. Negative supercoiled topoisomers are in white and positive supercoiled topoisomers in black. 2 μg/ml chloroquine introduces 14 positive supercoils. A white arrowhead indicates the topoisomer that migrated with ΔLk of 0 in the second dimension; it migrated with a ΔWr of –14 in the first dimension. A black arrowhead indicates the most abundant topoisomer. **(B)** pLS1 topoisomer distribution after different NOV treatments. Samples were taken before the addition of the drug (time 0 min) and at the times indicated. The corresponding supercoiling density (σ) value is indicated below each autoradiogram. Taken from Ferrándiz et al. (2010), with modifications.

The homeostatic control of supercoiling was first described in *E. coli.* In this bacterium, the transcription of *topA* (which codes for Topo I) was found to decrease under DNA relaxation (Tse-Dinh, 1985), while that of *gyrA*, and *gyrB* (which code for the two gyrase subunits) were found to increase (Menzel and Gellert, 1983, 1987a,b). An increase in gyrase expression in response to relaxation has also been observed in *Streptomyces* and *Mycobacterium* (Thiara and Cundliffe, 1989; Unniraman et al., 2002). However, in *Staphylococcus aureus*, treatment with NOV affects the transcription of the gyrase genes but not of *topA* (Schroder et al., 2014). In *S. pneumoniae*, treatment with NOV was also found to increase the transcription of gyrase genes, and diminish the expression of Topo I and Topo IV. In addition, global relaxation followed by a recovery of the native level of supercoiling was observed at low drug concentrations (Ferrándiz et al., 2010). The distribution of topoisomers in plasmid pLS1 (Stassi et al., 1981) was used to estimate the chromosomal superhelical density (σ), and returned a mean value of about -0.06 (**Figure 3**), which is within the range reported for the *E. coli* chromosome (Deng et al., 2005). At subinhibitory NOV concentrations (0.5× MIC), a transcriptomic response allowed the restoration of the native level of supercoiling after an initial relaxation causing a σ variation of 23%. A similar effect was observed at 1× MIC. However, higher concentrations of NOV increased the degree of relaxation with no further restoration of supercoiling, compatible with the saturation of the homeostatic capacity that results in the inhibition of cell division. The range of σ variation permitting homeostatic recovery of the supercoiling observed in *S. pneumoniae* is in agreement with the estimated ±20% variation compatible with normal cell growth in *E. coli* (Drlica, 1992). Supercoiling recovery in the pneumococcus occurred after the up-regulation of the gyrase genes *gyrA* and *gyrB* and the down-regulation of the Topo I (*topA*) and Topo IV (*parEC*) genes (Ferrándiz et al., 2010). In *E. coli*, the expression of the gyrase and Topo I genes is also mediated by NAPs, which affect DNA supercoiling (Travers and Muskhelishvili, 2005; Vora et al., 2009). However, these regulatory mechanisms may not function in *S. pneumoniae* for which NAP scarcity is predicted, and which certainly lacks most of the NAPs found in *E. coli*. Thus, supercoiling maintenance in *S. pneumoniae* appears to depend mainly on the regulation of topoisomerase transcription.

#### The Transcriptional Response to DNA Relaxation Involves Topology-Reactive Gene Clusters

**FIGURE 4:**
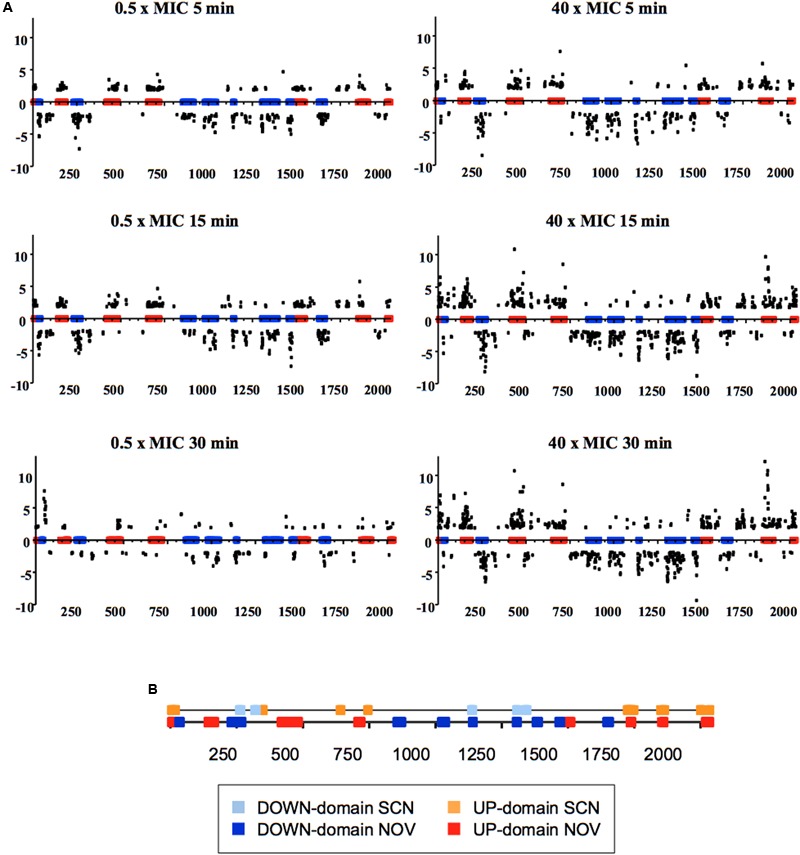
Global transcriptomic responses of *S. pneumoniae* to relaxation with NOV. **(A)** The relative fold variation of each gene is represented against the 3′ location of each open reading frame in the *S. pneumoniae* R6 chromosome (bases 1 to 2,038,615). Boxes indicate the transcriptional clusters: up-regulated in red, down-regulated in blue. **(B)** Localization of topological clusters detected as a result of treatment with either SCN or NOV. Taken from Ferrándiz et al. (2010, 2016a), with modifications.

The modulation of the expression of topoisomerase genes in *S. pneumoniae* is part of a global genome response (Ferrándiz et al., 2010). At subinhibitory concentrations, i.e., under physiological conditions, and short treatment times (5 and 15 min), DEGs were found to account for about 13% of the genome. An attenuation in the response at 30 min was observed, the number of DEGs being reduced to account for just 5.7% of the genome (**Figure 4A**), reflecting the recovery of supercoiling (**Figure 3**). Some 13% of the pneumococcal genome was therefore involved in the cellular response to moderate relaxation, allowing the recovery of the initial level of supercoiling. At fully inhibitory concentrations, the proportion of the genome covered by DEGs increased with time, from 14.4% at 5 min to 24% over longer periods (**Figure 4**). This agrees with the inhibition of cell division and with the continuous relaxation of the DNA (**Figure 3**). This proportion of the genome covered by DEGs upon relaxation is larger than in other bacteria. In Gram-negative bacteria, DEGs were found to account for 7% of the genome in *E. coli* [as determined using both gyrase inhibitors and gyrase thermosensitive mutants (Peter et al., 2004)], and for 8% in *Haemophilus influenzae* [as determined using NOV (Gmüender et al., 2001)]. In Gram-positive *Staphylococcus aureus*, treatment with NOV affected the transcription of 11% of the genome (Schroder et al., 2014).

It should be noted that the transcriptomic response to relaxation in *S. pneumoniae* involves topology-reactive gene clusters, or domains, that show coordinated up- or down-regulation. A total of 15 clusters have been detected, corresponding to 37% of the genome (**Figure 4**) (Ferrándiz et al., 2010). The sizes of these clusters varies from 14.6 to 85.6 kb (mean ± SD: 51.8 ± 21.8) and they contain 15–43 responsive genes (mean ± SD: 28 ± 9). They also include more than 68% of the DEGs. This has allowed topological clusters to be identified in which gene co-regulation is clearly more complex than would be expected simply from the number of genes in operons. In addition, the direction of transcription of the DEGs showed no preference for leading or lagging strands, providing additional evidence that topological control is structurally dependent.

The AT content over the genome correlates with domain location, and is higher in up-regulated (UP) than in down-regulated (DOWN) domains. These results suggest that the relaxation of DNA in AT-rich (ATr) regions favors the access of RNA polymerase to their promoters. On the contrary, a low AT content in DOWN clusters obstructs the access of RNA polymerase. Enrichment in the AT content of the region from positions -800 to +200 of genes up-regulated under relaxation has been reported in *E. coli* (Peter et al., 2004).

**FIGURE 5:**
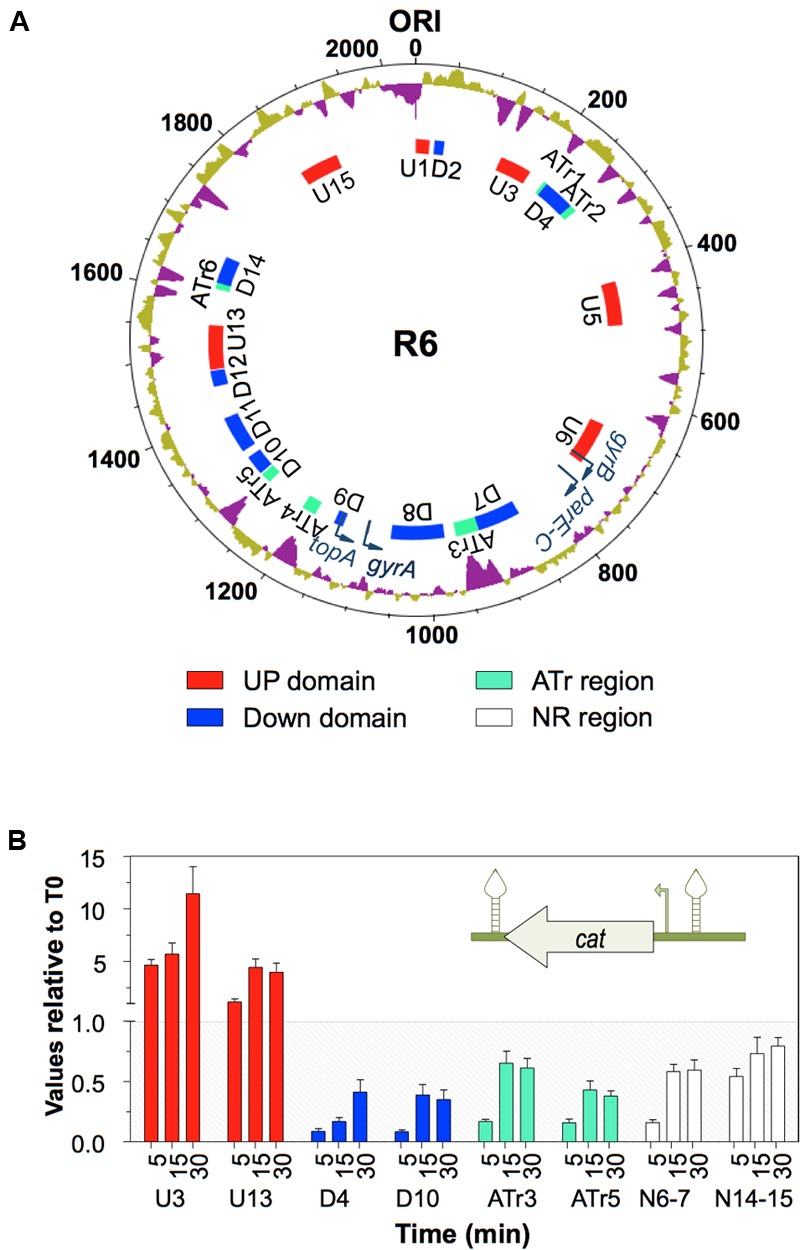
The topology-dependent transcription of P*_c_cat* is dependent on its chromosomal location. **(A)** Organization of the *S. pneumoniae* R6 chromosome in topological domains. Circles, from outside to inside, represent: % GC (values above the average in purple); DNA topoisomerase genes (dark blue curved arrows); topology-responsive domains. The chromosome is organized into domains up-regulated (U, red boxes) or down-regulated (D, blue boxes) in response to DNA relaxation, and ATr domains (green boxes). **(B)** Transcriptional response to DNA relaxation by NOV measured by qRT–PCR. A P*_t_*_c_*cat* cassette, coding for chloramphenicol-acetyl-transferase, which carries its own promoter (curved arrow) and is flanked by two transcriptional terminators (stem and loop structures), was inserted into different supercoiling domains. Cultures of the R6-CAT strains were treated with NOV and the transcription of *cat* analyzed by qRT–PCR. Taken from Ferrándiz et al. (2014), with modifications.

The organization of the *S. pneumoniae* chromosome into domains was further confirmed by the introduction of a *cat* heterologous gene cassette into the different types of domain (**Figure 5A**) (Ferrándiz et al., 2014). In response to relaxation with NOV, the transcription of *cat* was dependent on its chromosomal location, being up-regulated when located in UP domains, down-regulated when located in DOWN domains, and showing almost no changes when located in the non-regulated (NR) domains (**Figure 5B**). This all supports the idea that the chromosome is organized into topological domains that are reactive to interference in the supercoiling status. These results contrast, however, with those obtained in *E. coli*, in which the 306 DEGs were not only functionally diverse but widely dispersed throughout the chromosome (Peter et al., 2004), and with results obtained for *Staphylococcus aureus*, in which NOV-responsive genes were randomly distributed throughout the chromosome (Schroder et al., 2014).

### Response to Hypernegative Supercoiling Caused by the Inhibition of Topo I

The negative supercoiled state is the natural state of DNA homeostatic equilibrium in many bacteria. However, hypernegative supercoiling has been reported in *E. coli topA* mutants. With the exception of the *topA10* mutant, all have acquired compensatory mutations in the gyrase genes (DiNardo et al., 1982). The *topA10* mutant shows a notable 22% increase in negative supercoiling (Pruss et al., 1982), which probably represents the limit viable cells can afford in the long term. The inhibition of Topo I would produce greater hyper-supercoiling. Topo I plays an essential role in transcription, given its physical interaction with RNA polymerase (Cheng et al., 2003). During transcription, hypernegative supercoiling occurs behind the RNA polymerase, leading to RNA-DNA hybrid (R-loop) stabilization (Drolet, 2006). Topo I relaxes this supercoiling and prevents R-loop formation (Drolet et al., 1994; Phoenix et al., 1997; Masse and Drolet, 1999), allowing transcription to continue. Thus, the effects of hypernegative supercoiling in transcription depend directly on the activity of Topo I.

**FIGURE 6:**
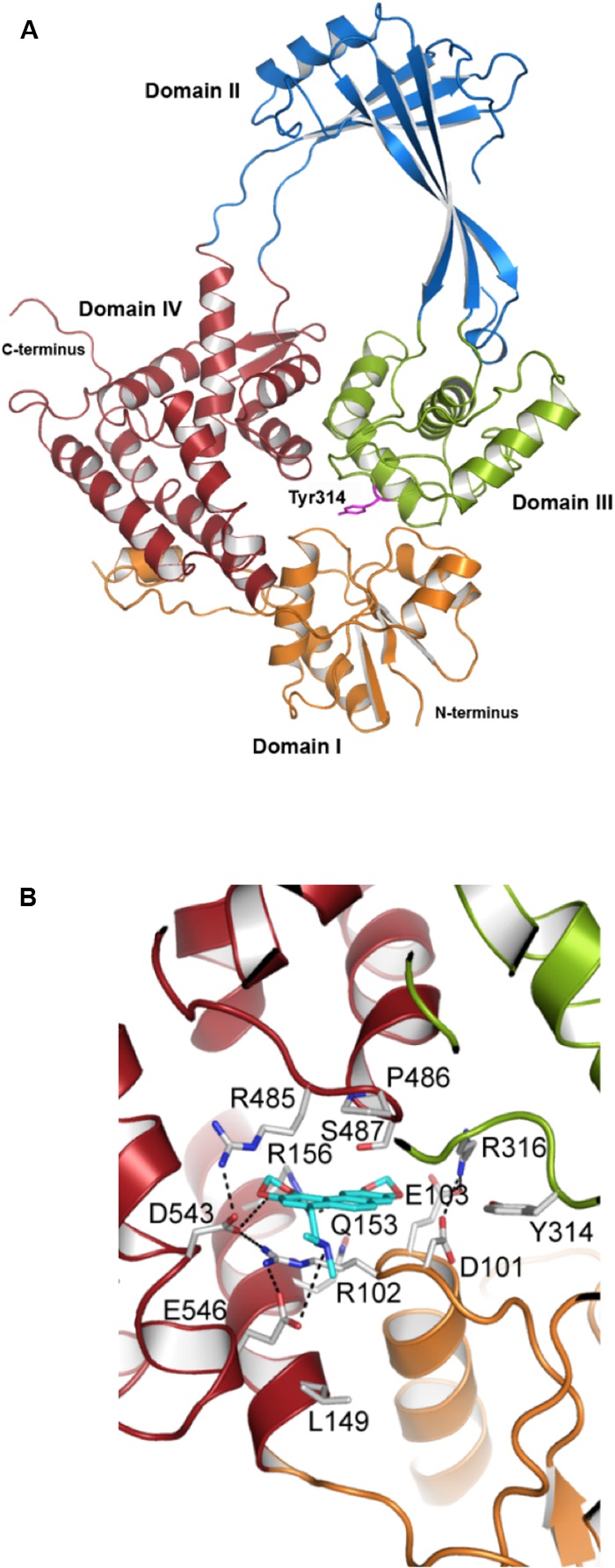
Structural modeling of the interaction of *N*-methyl SCN with *S. pneumoniae* topoisomerase I. **(A)** Modeling of the 67 kDa fragment of Topo I, showing domains I–IV and the catalytic Tyr314. **(B)** *N*-methyl-SCN (in blue) bound to the nucleotide-binding site of Topo I. Hydrogen bonds and salt-bridge interactions are indicated by dashed lines. Taken from García et al. (2011), with modifications.

However, Topo I-targeting compounds are extremely scarce. Cheng et al. (2007) identified an alkaloid, which, although it inhibits the activity of *E. coli* Topo I, did not inhibit cell growth significantly. Our group discovered a new inhibitor of *S. pneumoniae* Topo I, SCN, which inhibits its relaxation activity at concentrations equivalent to those that inhibit cell growth. The modeling of pneumococcal Topo I, based on the crystal structure of the *E. coli* enzyme (**Figure 6**), and docking to SCN, revealed strong interactions between the drug and the DNA-binding site of Topo I to correlate with the inhibitory effect observed (García et al., 2011).

**FIGURE 7:**
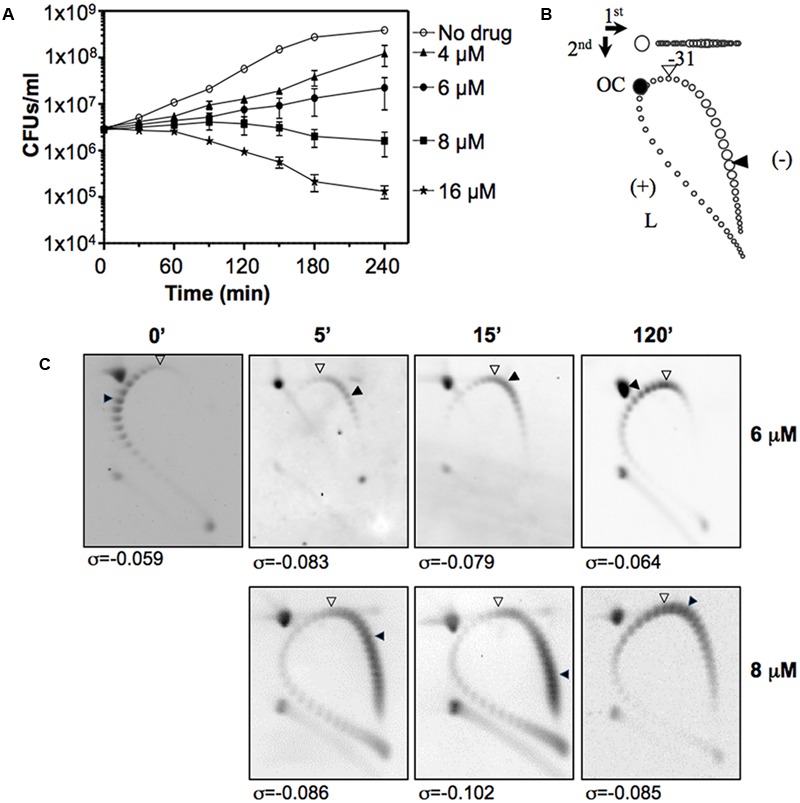
Seconeolitsine affects cell viability and induces hypersupercoiling. **(A)** Viability of R6 (pLS1) in a medium containing different SCN concentrations. Samples were taken before the addition of the drug (zero time), and at the indicated times were plated on drug-free agar medium. **(B)** Diagram showing topoisomer distribution in plasmid pLS1 subjected to 2D-agarose gel electrophoresis run in the presence of 5 and 15 μg/ ml chloroquine in the first and second dimensions, respectively. OC, open circle; L, linear form. A white arrowhead points to the topoisomer migrating with a ΔLk of 0 in the second dimension; it had a ΔWr of –31 in the first dimension. A black arrowhead points to the more abundant topoisomer. **(C)** Distribution of pLS1 topoisomers in 2D-gels after treatment with the indicated SCN concentrations. The supercoiling density (σ) values are indicated. Taken from Ferrándiz et al. (2016a), with modifications.

**FIGURE 8:**
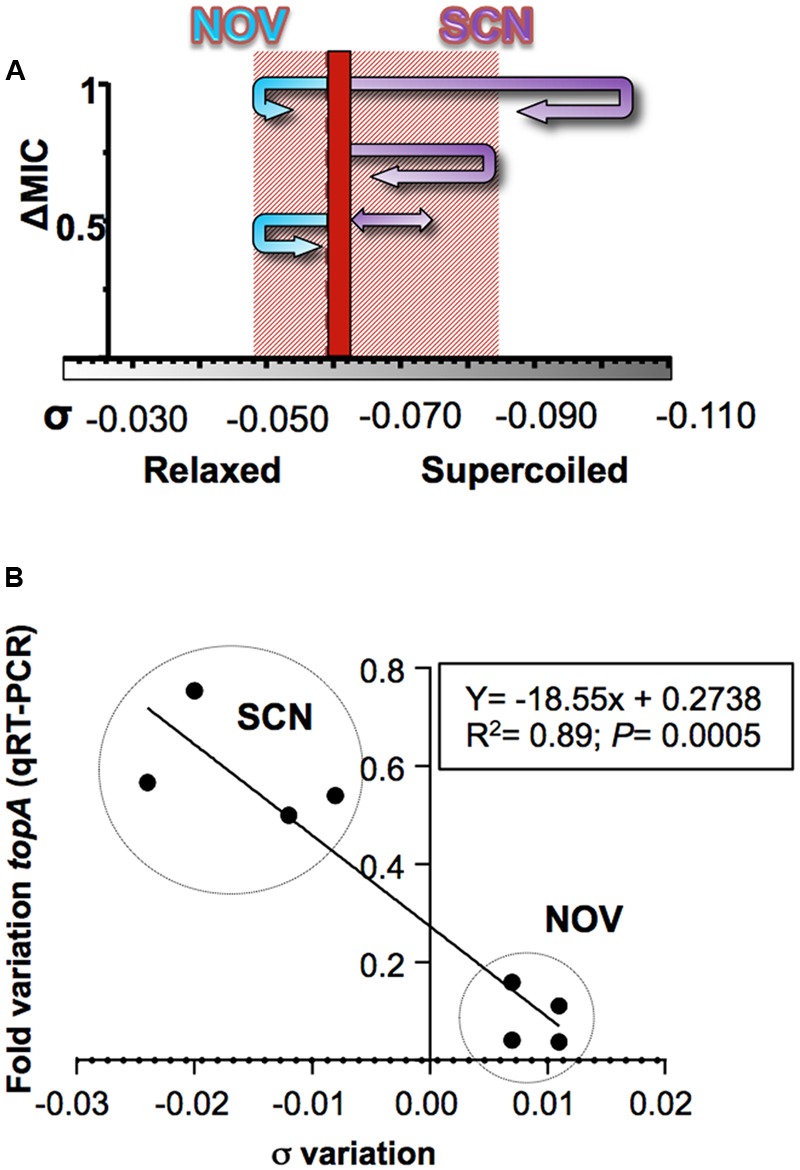
Responses to changes in supercoiling are conducted by Topo I. **(A)** Diagram showing the trajectory of the supercoiling density at the indicated NOV and SCN concentrations. A dashed line indicates the supercoiling density of DNA of non-treated cells. The shaded area represents the supercoiling density interval in which cells can survive. **(B)** Correlation between changes in supercoiling level and the transcription of *topA*. The data correspond to samples treated with either SCN or NOV at concentrations that allowed cell growth and the recovery of DNA supercoiling. Taken from Ferrándiz et al. (2016a), with modifications.

Our group was the first to use SCN in studies of the transcriptomic response to hypernegative supercoiling in bacteria (Ferrándiz et al., 2016a). The viability of *S. pneumoniae* and the increase in supercoiling is affected by SCN in a concentration-dependent manner (**Figure 7**). Treatment with 6 μM SCN produced a peak σ increase of 41% at 5 min, which later recovered. Treatment with 8 μM SCN resulted in higher and longer lasting increases in the σ value, with partial recovery after 120 min. These results show that treatment with subinhibitory SCN concentrations permit the recovery of peak σ increases of up to 41% without affecting cell viability. This tolerance to increases in supercoiling levels is greater than the 25% observed for DNA relaxation upon NOV treatment (**Figure 8A**) (Ferrándiz et al., 2010), and indicates that *S. pneumoniae*, and very likely genetically related bacteria, are naturally more tolerant to hyper-negative supercoiling than to hyper-relaxation. Similarly, the results of experimental evolution assays with *E. coli* revealed increasing supercoiling (associated with mutations in *topA*) to increase bacterial fitness (Crozat et al., 2005). A similar homeostatic mechanism allowing increased negative supercoiling might also exist in bacteria with reverse gyrase. These bacteria keep DNA in a slightly overwound state to protect their genome from heat damage (Ogawa et al., 2015).

The transcription levels of *topA* in *S. pneumoniae* at subinhibitory concentrations of SCN or NOV (which allow for cell growth and the recovery of supercoiling) show a good correlation with the induced variation in σ (**Figure 8B**). The regulation of *topA* therefore plays a fundamental role in the recovery of supercoiling levels. The variations seen in *topA* expression were, however, only part of a global transcriptomic response. Treatment with subinhibitory concentrations of SCN (8 μM, 0.5× MIC) generated a two-stage transcriptomic response: (i) early response and (ii) recovery. The former, which represents an active response against sharply increased supercoiling, was observed at 5 and 15 min of treatment, and involved about 11% of the genome. During recovery, only about 2% of the genome was involved at 30 min. In the early response, transcriptional variations also occurred in clusters, with DEGs grouping into topologically sensitive domains. The average size of a SCN cluster is 14.0 ± 7.6, similar to the 10 kb *E. coli* domains predicted using transcriptional data (Postow et al., 2004). Although the NOV and SCN clusters are not identical, their position in the chromosome nearly overlap (**Figure 4B**) – an unexpected finding given the opposing nature of DNA relaxation and supercoiling. These results support the idea that the chromosome is divided into topological domains with fixed locations.

### Regulation of DNA Topoisomerase Gene Transcription

In *E. coli*, several NAPs are involved in the regulation of topoisomerases. One such NAP is the FIS protein, which regulates the expression of genes coding for the subunits of gyrase (Schneider et al., 1999), Topo I (Weinstein-Fischer and Altuvia, 2007), and the genes coding for other NAPs involved in DNA supercoiling (Claret and Rouviere-Yaniv, 1996; Falconi et al., 1996; Grainger et al., 2008). In addition, two further NAPs, FIS, and H-NS proteins control both the level of supercoiling and global transcription (Blot et al., 2006; Marr et al., 2008). The corresponding situation in *S. pneumoniae*, which lacks these NAPs, seems to be much simpler.

The transcription of *gyrB* and *topA* in *S. pneumoniae* is regulated by their strategic chromosomal location in topological domains, since the expression driven by their promoters differs whether they are located in their natural chromosomal locations or in a replicating plasmid (Ferrándiz et al., 2014). Transcriptional fusions of these promoters to a reporter gene in plasmid pLS1 have been measured after DNA relaxation induced by NOV. As expected, relaxation caused down-regulation of *topA* and up-regulation of *gyrB* when the genes were located in their native chromosomal sites (DOWN9 for *topA* and UP6 for *gyrB* in **Figure 5A**). However, transcription from both promoters in the plasmid fusions was down-regulated. These results indicate that both *topA* and *gyrB* are under supercoil-mediated regulation, and that the plasmid behaves as a DOWN domain. This may serve to neutralize the high copy number of the plasmid genes and/or favor their replication.

**FIGURE 9:**
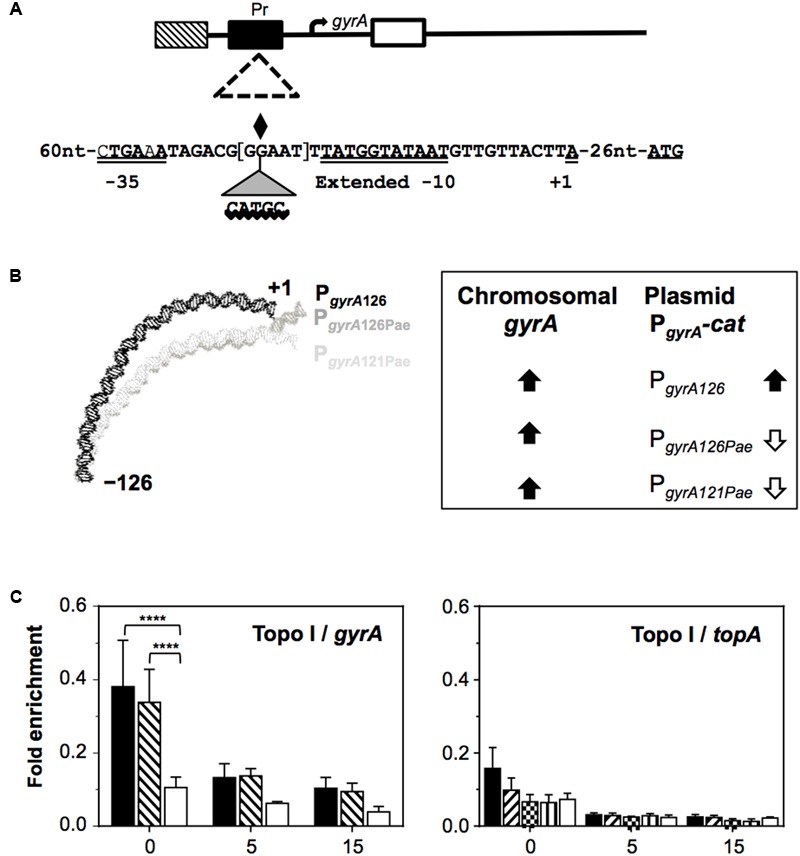
Control of the transcription of DNA topoisomerase genes by supercoiling. **(A)** Representation of the *gyrA* coding region and of the regions tested in chromatin immunoprecipitation, showing the sequences of the wild-type P*_gyrA126_*, P*_gyrA_*_126Pae_, and P*_gyrA_*_121Pae_ derivatives. The –35 and extended –10 boxes, the nucleotide at which transcription is initiated (+1), the center of the intrinsic DNA curvature (diamond), and the location of the inserted CATGC sequence that creates a *Pae*I restriction site, are all indicated. The five nucleotides deleted in P*_gyrA_*_121Pae_ are in brackets. **(B)** The relaxation-induced up-regulation of P*_gyrA_* depends on intrinsic bending: curvature prediction and results obtained from qRT-PCR analysis. **(C)** Recruitment of Topo I to *topA* and *gyrA* upstream sequences. Exponentially growing cells were subjected to chromatin immunoprecipitation using anti-Topo I antibodies; the pulled-down DNA was subsequently analyzed by qPCR. The graphs show the pulldown efficiency (ChIP-DNA/input DNA) for each primer pair. Values are the average ± SD of three independent replicates. ^∗∗∗∗^*P* < 0.0001. Taken from Ferrándiz et al. (2014, 2016a), with modifications.

In contrast, the Topo IV genes (*parE* and *parC*) and *gyrA* are located in NR domains, and their expression depends on specific regulatory signals located in the promoter region. The expression of the Topo IV genes from their common promoter (Balsalobre and de la Campa, 2008) is equivalent in their natural chromosomal location and in plasmids (Ferrándiz et al., 2014). With respect to the *gyrA* gene, its upstream region (P*_gyrA_*_126_, nt -126 to +1 in **Figure 9A**) shows an intrinsic DNA curvature (Balas et al., 1998). This was fused to *cat* and cloned into plasmid pLS1, and the curvature either eliminated by a 5 bp insertion (P_gyrA126Pae_) or by a 5 bp deletion (P_gyrA121Pae_), and a direct correlation observed between *cat* expression and the curvature under basal conditions (the specific activity of the P_gyrA126_ fusion was ∼3-fold higher than that recorded for plasmids lacking the curvature). This shows that the curvature behaves as an activator *per se*, providing better recruitment of either the RNA polymerase complex or specific regulatory proteins. The role of curvatures as regulators of transcription has previously been established in bacteria (Pérez-Martín et al., 1994), including *S. pneumoniae* (Pérez-Martín and Espinosa, 1991). In addition, the transcription levels from the chromosomal P*_gyrA_* and the P*_gyrA_cat* fusions in plasmids in the presence of NOV have been determined. While in the plasmid carrying the wild-type promoter (P_gyrA126_) the up-regulation of *cat* was similar to that of the chromosomal *gyrA*, down-regulation of *cat* was observed in the plasmids lacking the curvature (**Figure 9B**). These results suggest that the signals regulating *gyrA* transcription are included within the above-mentioned 126 nt region, and that bending is a key element for its regulation under relaxation by acting as a sensor of the supercoiling level.

Chromatin immunoprecipitation experiments using antibodies directed against the pneumococcal GyrA subunit and Topo I (Ferrándiz et al., 2016a) have shown P*_gyrA_* to recruit Topo I, but not gyrase (**Figure 9C**). The region to which Topo I binds includes the -35 and extended -10 boxes on P*_gyrA_*, plus the DNA curvature (Balas et al., 1998). Thus, Topo I, the transcription of which is regulated by supercoiling levels, appears to be the key factor regulating *gyrA* expression.

## Evolutionary Pressure Drives the Organization of the Chromosome into Domains

### Domain Conservation in Streptococci

**FIGURE 10:**
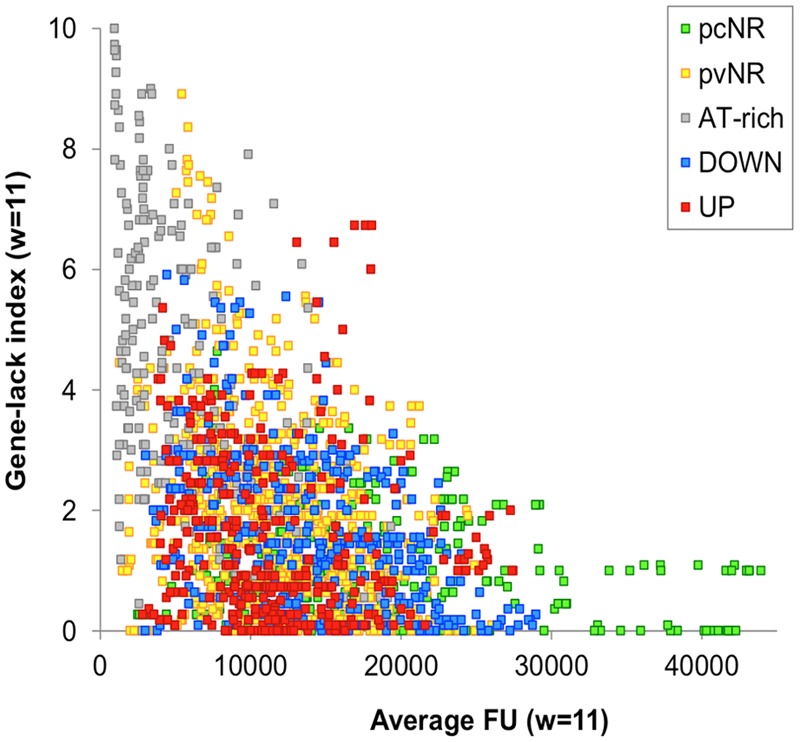
Evidence for conservation of UP and DOWN domains in *S. pneumoniae*. Relationship between the gene-lack index and gene expression (fluorescent units, FU) in *S. pneumoniae* R6 as detected in high density microarrays. A total of 12 genome sequences (from 11 clinical isolates and R6) were analyzed. Genes of clinical isolates were considered equivalent to those of R6 when their products shared ≥80% similarity over ≥80% of the sequence length. An 11-gene window (about 10 kb) was contemplated.

**FIGURE 11:**
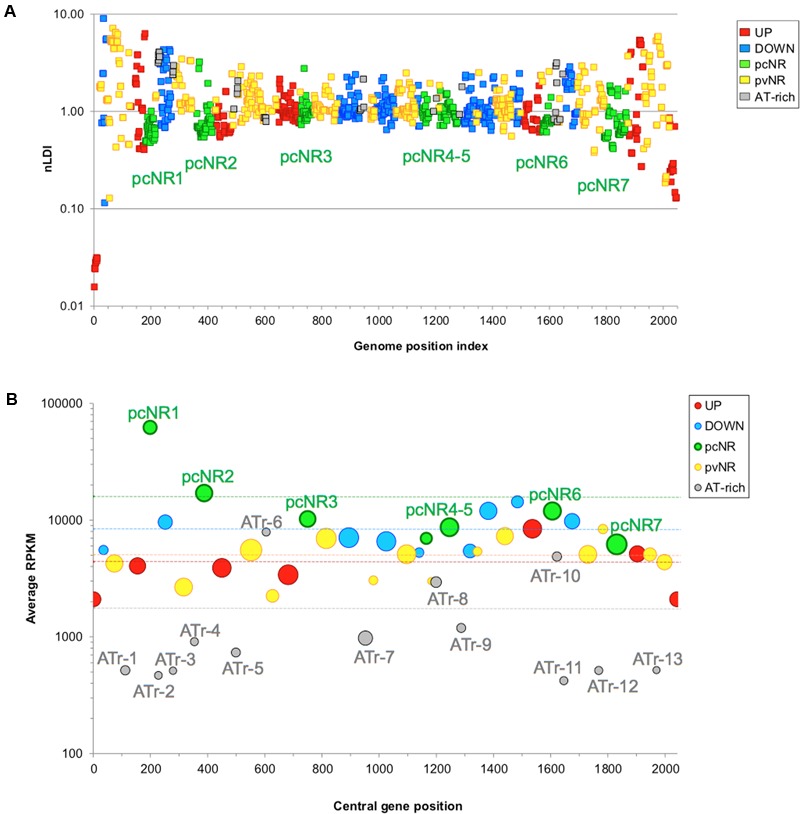
Different domains have different locations and transcription levels. **(A)** Location of NOV-reactive and pcNR domains. Dots indicate nLDI values for *S. pneumoniae* R6 genes for the whole *Streptococcus* genus. A total of 1216 genes coding for proteins in *S. pneumoniae* R6, with homologs in ≥10 species, were contemplated in the analysis. Gene indexing starts from the replication origin. **(B)** Abundance of RNA transcripts according to domain type. RNA-Seq data were normalized by gene size (kb) using the reads per kb per million mapped reads (RPKM). The positions of the domains in the genome are represented by the position of the central gene (central gene position, *X*-axis). The size of the circles is proportional to the number of genes in the domain. Only pvNR clusters with at least 10 members are shown. Horizontal lines in the corresponding color indicate the average RPKM value for each domain class. Taken from Martín-Galiano et al. (2017).

Gene order in bacterial chromosomes surpasses the level of the operon (Lathe et al., 2000; Reams and Neidle, 2004). As explained above, and based on its transcriptome under DNA relaxation, the chromosome of *S. pneumoniae* R6 appears to be organized into four types of topological domains: UP, DOWN, NR, and ATr. The analysis of 12 *S. pneumoniae* complete genome sequences has revealed the conservation of the UP and DOWN domains (**Figure 10**). The gene-lack index (number of genomes in which a gene is absent divided by the total number of genomes) revealed lower values for the UP (1.51) and DOWN (1.65) domains than the genome average (1.91). However, ATr domains have high gene-lack indices (average 4.66), suggesting extensive gene interchange in these domains. To study the conservation of domains, normalized location dispersion indices (nLDI: values that quantify the position deviation of a given gene with respect to the Ori, and relative to homologs in several genomes (Martín-Galiano et al., 2017)) were calculated across *S. pneumoniae* genomes; the values returned were very small since synteny is highly conserved in this species. The same was then calculated for representative strains of 25 species of *Streptococcus* in order to detect distinguishing differences. The conservation of *S. pneumoniae* domains across these *Streptococcus* representatives was then determined. Two assumptions were made: (i) that the gene order is relatively conserved, as seen in gamma-proteobacteria (Sobetzko et al., 2012), and (ii) that chromosomal topology is conserved, given that species share core gene pools (Lefebure and Stanhope, 2007), similar genome lengths, and a similar AT content. Similar approaches have been followed to examine chromosomal patterning in other bacteria (Wright et al., 2007; Khedkar and Seshasayee, 2016). In *S. pneumoniae*, 571 genes (28.0%) had nLDI values of <1, which indicates they tend to locate to positions more stable than the average for maintained homologs (Martín-Galiano et al., 2017). Several genes from the UP and DOWN domains were present in most streptococci at equivalent positions. The greatest position conservation was observed in 40 genes near the Ori, indicating strong topological pressure to maintain functionalities in this region. Genes near the Ori have high copy numbers (Slager and Veening, 2016) and show a peculiar pattern of NAP binding (Sobetzko et al., 2012). Moreover, seven clusters with conserved positions were detected for NR genes, and named pcNR domains (position-conserved Non-Regulated domains). Most of the remaining NR genes were organized into 14 domains (≥10 genes) termed pvNR domains (position-variable Non-Regulated). ATr regions accounted for 13 domains (**Figure 11**). Strikingly, the pcNR domains appeared symmetrically located at regular intervals (∼200, 400, and 800 kb) on both sides of the Ori and were interleaved between UP, DOWN, and pvNR domains (**Figure 11A**). The size of these domains appeared compatible with the 100 kb lengths estimated for them using different techniques (Worcel and Burgi, 1972; Sinden and Pettijohn, 1981; Le et al., 2013). This suggests a potential higher-order macrostructural unit above the domain level controlling the genetic stability and plasticity required to face new environments (Rocha, 2004a).

### Levels of Protein Expression and Essentiality of the Domains

The transcriptomes of exponentially growing cultures (Ferrándiz et al., 2016a,b) showed the pcNR domain transcription level to be higher than that of the ATr domains (**Figure 11B**). Two factors contribute to these transcriptional differences. First, long repeat sequences (BOX, RUP, and SPRITE) (Croucher et al., 2011), which are associated with the repression of transcription, are few in pcNR domains, and second, the codon adaptation index (CAI), which is related to the translation rate and mRNA levels (Martín-Galiano et al., 2004), is high in pcNR domains (Martín-Galiano et al., 2017). Gene location also affects protein levels (Ochman et al., 2000; Rocha, 2004b), a pattern associated with the distance to the Ori. Genes at the Ori are doubly represented with respect to genes at the Ter in *E. coli* during exponential growth (Chandler and Pritchard, 1975). Accordingly, the relocation of genes coding for ribosomal proteins and the RNA polymerase alpha subunit to positions distant to the Ori, reduces their transcription rates, which was associated with slower growth in *Vibrio cholerae* (Soler-Bistue et al., 2015). Similarly, in *Salmonella typhimurium*, genes relocated near the Ori are expressed more strongly than those relocated near the Ter (Schmid and Roth, 1987). The regular positioning of strongly expressed genes may mark the limits of domains, as reported for *Caulobacter crescentus* (Le et al., 2013).

**FIGURE 12:**
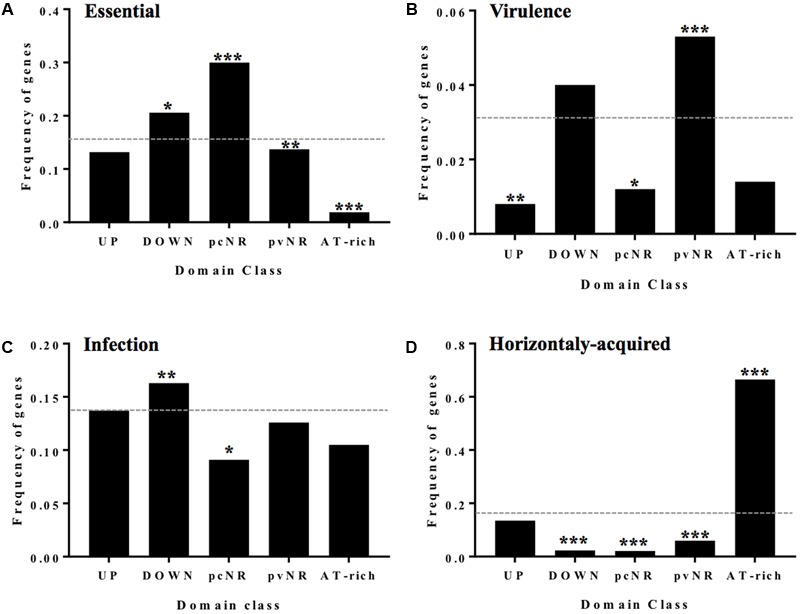
Genes of the different domains have different functionalities. **(A)** Fraction of essential genes in different domains. **(B)** Virulence factors. **(C)** Horizontally acquired genes abundance and domain class. **(D)** Essential genes for infection by STM. Statistical significance with respect to the genome average (dashed line): ^∗^*P* ≤0.05, ^∗∗^*P* ≤ 0.01, ^∗∗∗^*P* ≤ 0.001. Taken from Martín-Galiano et al. (2017), with modifications.

The fraction of essential genes, as determined by Tn-seq (van Opijnen and Camilli, 2012), is notably higher in pcNR domains than in the other domains (**Figure 12A**). The co-localization of essential genes beyond randomness has also been reported for *Bacillus subtilis* and *E. coli* (Fang et al., 2005), perhaps because clustering makes genomes more resistant to deletions (Fang et al., 2008). The number of pcNR genes in the lagging strand was 15.6%, significantly lower than the average in the remaining *S. pneumoniae* genome (22.3%). This would reduce the chances of collision between DNA and RNA polymerases, resulting in the discontinuation of transcription (French, 1992). Essential genes also tend to be more strongly expressed (Rocha and Danchin, 2003), as confirmed for pneumococcal pcNR genes. Essential gene clustering at regular intervals, and not affected by topological stress as defined for pcNR, appears to reflect a favorable “supercoiling environment” for protein expression.

### The Different Domains Contain Genes with Different Functions

#### Importance of the Protein Interaction Network

A significant fraction of the pcNR genes codes for proteins with important roles in central metabolism and that have a high number of protein–protein interactions (PPIs). PPIs provide a rough estimate of a protein’s importance in cell physiology. The estimated amounts of protein produced, and their functions, support the idea that the genes of pcNR domains are more involved in the central metabolic network than are those of the pvNR domains. In stark contrast, ATr genes appear to play little or no role in central metabolism; their PPI values are at most only about one third of the average for the remaining genome. As mentioned above, changes in the location of genes could lead to alterations in cell physiology, which holds true for both central metabolic (Soler-Bistue et al., 2015) and regulatory genes (Gerganova et al., 2015). The physical positioning of specific supercoiling-favorable regions in the chromosome is also related to the ability to gain access to cytoplasmic regions rich in ribosomes (Soler-Bistue et al., 2015).

Overall, the evidence supports the idea that the function, expression, essentiality and stability of genomic positions are interconnected, as reported for *Dickeya dadantii* and *E. coli* (Sobetzko et al., 2012; Jiang et al., 2015). Altogether, the pcNR genes reflect a multistep adaptation in the transcription-translation-interaction cascade that facilitates the activity of these genes’ products, thereby increasing bacterial fitness.

#### Pathogenesis and Immunogenicity

DNA topology regulates the expression of virulence factors in several bacteria (Dorman and Porter, 1998; Cameron and Dorman, 2012; Reverchon and Nasser, 2013; Jiang et al., 2015). In *S. pneumoniae*, three types of virulence genes show differences in their distribution among domains. Widely accepted virulence factors are more abundant in pvNR domains (**Figure 12B**), while genes contributing [as estimated by signature-tagged mutagenesis (Hensel et al., 1995)] to intranasal colonization, meningitis or otitis (Chen et al., 2008; Molzen et al., 2011) are more abundant in DOWN domains (**Figure 12C**). Finally, genes coding for proteins that trigger an immune response in humans (Giefing et al., 2009), and which are therefore candidate targets for a serotype-independent protein-based vaccine against pneumococcus, are predominant in the pvNR domains. The pvNR domains also contain more genes coding for extracellular proteins or proteins anchored in the cell wall than do pcNR domains. All in all, pvNR domains show strong allelic variation by being subjected to selective pressure during adhesion, cytotoxic challenge and immune system evasion. This variation also increases the genome pool of the species via gene duplication/paralogs in which one copy is not subject to immediate pressure (Mira et al., 2010). The link between supercoiling stress and virulence enhancement does not seem to be the rule for *S. pneumoniae*, the canonical virulence and accessory factors of which are preferentially encoded in the pvNR or DOWN domains.

#### Genes Involved in Competence

Gene transfer is a primary driver of evolution in bacteria, but the introduction of new genetic material at random can perturb chromosomal topology. *S. pneumoniae* is a naturally transformable bacterium (Claverys et al., 2006; Martin et al., 2006), the evolution of which (including its antibiotic-resistance and virulence factors) depends on both intra-species and inter-species chromosomal transformation (Dowson et al., 1989, 1990; Balsalobre et al., 2003; Ferrándiz et al., 2005). Competence involves the transient transcriptional modulation of ∼10% of the genome with strict timing (Peterson et al., 2004). When under stress (the X state), the competence system -which bears some resemblance to the SOS repair system of *E. coli* and other bacteria – is activated (Claverys et al., 2006). In fact, FQs induce the SOS response since they cause double-strand breaks in chromosomes (Drlica et al., 2008). As described above, local supercoiling changes triggered by FQs activate pneumococcal competence, but global supercoiling changes do so too. The early and delayed up-regulated competence genes (those activated during stress) are mainly located in UP domains. Many pcNR genes are, however, down-regulated, indicating that during the X-state the topology of the chromosome is perturbed to a degree that threatens cell viability via effects on the central metabolic machinery. This explains why growth is slowed during competence (Oggioni et al., 2004) and why several mechanisms have been acquired, including the use of small untranslated RNAs and proteases to actively terminate the X-state and promptly recover the normal topological situation (Echenique et al., 2000; Cassone et al., 2012).

#### Horizontally Acquired Genes

In *S. pneumoniae* R6, up to 12.1% of the genome is thought to have been acquired by horizontal gene transfer. The distribution of these acquired genes among domains is uneven, with a clear bias toward ATr domains (**Figure 12D**). This suggests that these domains act as structural or parasitic DNA hotspots, which agrees with their low transcriptional level and annotated functions (Ferrándiz et al., 2010, 2014, 2016a). It remains open the possibility that the ATr regions influence the organization of topological dynamics, or that they are involved in the acquisition of foreign genes.

## Conclusion and Perspectives

The transcriptome of *S. pneumoniae* alters with local or global changes in supercoiling. Local changes induced by the clinically used FQs LVX, and MOX, which target GyrA and/or Topo IV, trigger a transcriptional response. Both FQs up-regulate the competence regulon in response to stress, and, respectively, cause an increase in intracellular ROS by increasing the uptake of iron (through up-regulation of the *fatDCEB* transporter) and hydrogen peroxide (through up-regulation of the glycolytic pathway), both of which are involved in the Fenton reaction.

Changes in global supercoiling induced by NOV (which targets GyrB), or by SCN (which targets Topo I), have revealed the existence of topological domains that react in a coordinated fashion. In *S. pneumoniae*, the control of DNA-supercoiling occurs mainly via the regulation of transcription of the topoisomerase genes: relaxation triggers the up-regulation of *gyrA* and *gyrB* and the down-regulation of the Topo I (*topA*) and Topo IV (*parEC*) genes, while hypernegative supercoiling triggers the down-regulation of *topA*. The transcription of *gyrB* and *topA* is regulated by their strategic chromosomal location in the topological domains, while the expression of *parEC* and *gyrA* depends on the specific regulation of their promoters. Although the regulators of *parEC* are unknown, the promoter of *gyrA* shows an intrinsic curvature that acts as a sensor of the supercoiling level. In addition, chromatin immunoprecipitation experiments have revealed Topo I to bind to the *gyrA* promoter. Therefore, Topo I, the transcription of which is regulated by the supercoiling level, appears to regulate *gyrA* expression.

The regulation of topoisomerase genes is part of a global response to changes in supercoiling. Relaxation affects >13% of the genome (from 13 to 24%), while hypernegative supercoiling affects 10%. In both cases, responsive genes are grouped into domains that essentially overlap, suggesting that they have a fixed chromosomal location. Based on their structural and functional characteristics, and the change in the domains detected under relaxation, the following types can be defined: UP, DOWN, pcNR, pvNR, and ATr. The genes of the UP, DOWN, and pcNR domains have been found at equivalent positions present in most streptococci, especially near the Ori. pcNR domains are interleaved between UP, DOWN, and pvNR domains, which suggests a higher-order macrostructural unit. The pcNRs genes show the highest level of transcription, and contain most of the essential genes plus those involved in the central metabolic network. In stark contrast, the ATr domains show the lowest transcriptional levels, and the genes they contain appear to have little to do with the central metabolic network. This explains the tropism of pcNR genes for topologically secure areas, helping to maintain the constant provision of central proteins.

The genes coding for the classical virulence factors, plus those coding for immunogenic proteins, are more common in the pvNR domains, while genes contributing toward the establishment of infection are more common in the DOWN domains. The distribution of horizontally acquired genes is clearly biased toward ATr domains, suggesting these to be hotspots for the acquisition of foreign genes.

In general, UP gene expression is favored by topological stress; DOWN genes are highly expressed under favorable conditions and less so during such stress. ATr domains may sense topological stress and modify supercoiling in their area to reduce the transcription of adjacent genes, preferentially those in the DOWN domains. The chromosome supercoiling structure may act as a multi-sensor with homeostatic capacity, adapted to react to unfavorable conditions.

Pneumococcal genes appear to be subject to topology-driven selection that defines the chromosomal location of genes involved in metabolism, virulence and competence. Together, these organizational features reveal the genome of *S. pneumoniae* to be influenced by physiology-related topological rules. A global topology theory might be envisaged in which gene positioning is far from random. Many aspects of the importance of gene location – such as the idiosyncrasy of the domains and how this affects fundamental aspects of bacterial biology – are only now becoming understood.

Topological genomics – topogenomics – provides an alternative paradigm of genome analysis. Certainly, genome architecture plays an important role in the pathobiology and evolution of *S. pneumoniae*, and it is tempting to speculate that in other species too, the genes are subjected to topology-driven selection pressure that defines their chromosomal locations. Data from many species will, however, be needed before a full understanding of all the rules underlying topogenomics are known and understood.

## Author Contributions

All authors made intellectual contributions to the work and approved it for publication. AdC supervised all the studies and wrote the manuscript. MF performed most of the experiments related to determinations of supercoiling densities and transcriptomic studies. AM-G performed the bioinformatic studies. MG performed the characterization of topoisomerase I and its inhibition by seconeolitsine. JT-V contributed to the experiments of chromatin immunoprecipitation.

## Conflict of Interest Statement

The authors declare that the research was conducted in the absence of any commercial or financial relationships that could be construed as a potential conflict of interest.
